# Moisture Preparation and Bending Performance of Metal Plate Connected C24 Spruce Members in an Exploratory Comparison of Two Connector Systems

**DOI:** 10.3390/polym18192393

**Published:** 2026-09-30

**Authors:** Marek Wieruszewski, Weronika Magdziak, Arkadiusz Gronowski, Agnieszka Katarzyna Wdowiak-Postulak

**Affiliations:** 1 Department of Mechanical Wood Technology, Poznań University of Life Sciences, 60-637 Poznań, Poland; marek.wieruszewski@up.poznan.pl (M.W.); weronika.m4@gmail.com (W.M.); arkadiusz.gronowski@sodra.pl (A.G.); 2 SODRA POLSKA sp. z o.o., Gdyńska 15A, 62-028 Koziegłowy, Poland; 3 Department of Materials Strength and Structural Diagnostics, Kielce University of Technology, 25-314 Kielce, Poland

**Keywords:** metal-plate-connected joints, C24 spruce, moisture preparation, four-point bending, ultimate load, confounding, parent-beam variability, exploratory analysis

## Abstract

Moisture preparation, moisture at testing, and timber variability can jointly influence the response of metal-plate-connected (MPC) members. This exploratory re-analysis concerns 18 C24 Norway spruce assemblies (45 × 170 mm) with Mitek T150 or Ristek LL13 plates. The principal comparison includes 14 directly conditioned specimens in nominal 15%, 20%, and 40% preparation groups, with only two or three beams per connector–group cell; four moisture-transition specimens are described separately. Pooled mean ultimate loads were 14.15, 11.41, and 10.22 kN, respectively. An additive model summarized a nominal-group association (F(2,10) ≈ 12.29, *p* ≈ 0.002; bias-reduced, nonpartial ω^2^ ≈ 0.587), but this estimate remains uncertain in such a small sample. The connector comparison was inconclusive (*p* ≈ 0.122), rather than evidence of equivalent performance. In the nominal 40% group, reported mean moisture differed substantially between Mitek and Ristek (40.45% and 26.35%), and resistance-meter readings in the wet range lack documented validation. Separate verified moisture records at pressing and testing, and the timber-allocation procedure, are not documented. Consequently, preparation history, test-state moisture, connector system, and possible timber-batch differences cannot be separated causally. Laboratory coupons were summarized at the parent-beam level, without treating subsamples as independent treatment replicates. The findings identify associations requiring a balanced, replicated experiment; they do not establish an assembly-moisture effect, a connector ranking, or a design reduction factor.

## 1. Introduction

Prefabricated timber roof trusses are efficient lightweight structural systems in which the load path is strongly influenced by joint behavior. Punched metal connector plates transfer axial force, shear, and local bending through a dense field of teeth pressed into the timber; consequently, global truss stiffness cannot always be represented adequately by ideal pin joints. General timber-engineering texts and recent reviews emphasize that connector deformation, plate orientation, timber density, member geometry, and fabrication quality can be decisive for both serviceability and ultimate resistance [1,2,3,4].

Design and product-control frameworks therefore treat the timber member and the punched plate as a coupled system. Eurocode 5 [5], ANSI/TPI 1 [6], EN 14545 [7], and EN 14250 [8] govern structural verification, connector properties, and prefabricated truss production, while EN 1075 [9] and EN 408 [10] provide relevant mechanical test procedures. Strength classification and moisture-related corrections are linked to EN 338 [11], EN 384 [12], and moisture determination to EN 13183-2 [13]. These documents do not remove the need for experimental characterization: the effective stiffness and failure mechanism of an MPC joint depend on the specific plate-to-wood interaction and the actual state of the timber.

Wood moisture content is a primary environmental variable because hygroscopic changes alter cell-wall stiffness, dimensional stability, and local bearing around fasteners. Gerhards showed that most clear-wood mechanical properties increase as moisture decreases below the fiber-saturation region, although sensitivity varies by property and test mode [14]. The Wood Handbook likewise treats moisture as a central modifier of strength, stiffness, dimensional response, and durability [15]. For MPC joints, this bulk wood response is compounded by highly localized tooth embedment, fiber crushing, friction, and possible plate backout.

Direct environmental studies on truss plates have demonstrated that the joint can be more moisture-sensitive than a simple dry-state strength comparison suggests. Groom reported that mild moisture cycling reduced truss-plate strength and stiffness more rapidly than constant-moisture exposure and that more severe cycling further accelerated deterioration [16]; a companion USDA study identified plate backout and loss of tooth–wood interfacial integrity as important mechanisms [17]. Mtenga et al. exposed metal-plated joints to showering, wet–dry sequences, and 24 h soaking and reported losses exceeding 40% of load-carrying capacity in wet conditions relative to dry controls [18]. These studies establish a mechanistic basis for treating moisture history, rather than only nominal service class, as a structural variable.

The underlying load-transfer behavior has been examined in tension, shear, combined loading, and joint-specific tests. Gebremedhin et al. quantified nonlinear load–slip response for multiple plate/grain orientations [19]; Gupta and Gebremedhin reported destructive strengths and failure modes for several truss-joint geometries [20]; Vatovec et al. measured translational and rotational stiffness of representative joints [21]; Cramer et al. developed a theoretical treatment of wood-splice joints [22]; and Ellegaard used finite-element models to reproduce connector response while identifying limits associated with cracking and nonlinear contact [23]. Collectively, these studies show why ultimate force, global bending stiffness, local plate slip, and tooth withdrawal should not be treated as interchangeable response variables.

At the system scale, semi-rigid connection models change member-force redistribution and predicted deflections. Gupta et al. demonstrated the relevance of semi-rigid joint representation in truss analysis [24]. Dynamic and short-term cyclic tests have further shown that MPC stiffness can degrade under large cyclic histories even when residual static strength is comparatively stable [25,26], while fatigue tests confirm that repeated loading may alter slip behavior [27]. Weathering, long-term humidity, creep, and service deflection have likewise been examined in connection and truss studies [28,29,30,31]. Thus, environmental and loading histories interact, and an experimental design must distinguish true moisture effects from seating, repeated loading, creep, and specimen-to-specimen timber variability.

A substantial body of foundational work also establishes the dependence of MPC performance on joint geometry and loading direction. Studies of light-frame joints include early analyses of plate effects on frame response, truss-plate modeling, pure bending and tensile strength, fatigue, plate orientation, combined bending–tension interaction, and purpose-built combined-load test methods [32,33,34,35,36,37,38,39,40,41,42]. Dynamic resistance and tension-joint stiffness/load distribution were subsequently quantified experimentally [43,44,45,46]. These studies consistently point to tooth withdrawal, plate tearing/yielding, local wood crushing, and splitting as competing failure mechanisms whose prevalence changes with connector geometry and the state of the wood.

Model development has progressed from nonlinear tooth-on-foundation formulations to detailed three-dimensional and contact-based finite-element representations. Groom and Polensek modeled nonlinear tooth–wood transfer [47]; Vatovec et al. developed a spring-based three-dimensional joint model and extended it to a complete truss [48,49]; Amanuel et al. represented the tooth–wood interface with contact elements [50]; and Cabrero and Gebremedhin predicted tension-splice and heel-joint stiffness with explicit slip and friction [51]. These methods demonstrate that “joint stiffness” is a local constitutive property, whereas a modulus back-calculated from full-beam deflection is an apparent global property containing timber bending, plate slip, rotation, seating, and measurement effects.

More recent work has extended MPC research to nontriangulated openings, assembly stability, scaled modeling, strengthening, high-fidelity buckling analysis, temperature exposure, and probabilistic robustness [52,53,54,55,56,57,58,59,60]. In particular, current tests of punched plates continue to report substantial connector ductility and measurable translational/rotational stiffness [60], while modern stability analyses explicitly identify plate restraint as a boundary condition rather than an ideal hinge [6,7,8,9,10,11,12,13,14,15,16,17,18,19,20,21,22,23,24,25,26,27,28,29,30,31,32,33,34,35,36,37,38,39,40,41,42,43,44,45,46,47,48,49,50,51,52,53,54,55,56,57,58,59,60,61,62]. This reinforces the need to analyze full-size flexural tests with appropriate statistical caution.

The present analysis concerns a separate 18-member industrial test campaign, not the repeated wetting–drying experiment reported by Wieruszewski et al. [2]. Its purpose is to describe differences in ultimate load among nominal preparation groups and two connector systems, and to examine how parent-material variability limits their interpretation. The comparison is exploratory: moisture at pressing and moisture at testing were not independently varied in a documented factorial design, and random allocation of timber is not established. We therefore distinguish observed group associations from causal effects, retain the four transition histories as individual cases, and evaluate the laboratory coupons at the parent-beam level. Previously tabulated apparent global moduli are retained only as a measurement diagnostic in Appendix A.

## 2. Materials and Methods

### 2.1. Structural Timber and Connector Systems

The experimental material comprised strength-graded C24 Norway spruce (Picea abies) structural timber from Scandinavian supply. Full-size members had a nominal cross-section of 45 × 170 mm and consisted of two timber pieces joined end-to-end. The available description gives an overall specimen length of approximately 2600 mm {component dimensional allowance}, but also gives approximately 1200 mm for each component; these dimensions are inconsistent and the component lengths cannot be established from that description. The reported 2550 mm support span is retained as the test-geometry value, subject to verification against the test records. C24 denotes the input timber grade, not a strength classification established for the spliced assemblies [11].

Two commercial punched metal plate systems were compared: Mitek T150 (MiTek Industries Polska Sp. z o.o., Legnica, Poland) and Ristek LL13 (Ristek Oy, Villähde, Finland). Both were galvanized steel products intended for structural timber trusses and were applied symmetrically to the joint. Table 1 lists the reported plate dimensions, steel grades, and sheet thicknesses. These are product descriptions, not measurements made in the present re-analysis. Because these characteristics differ simultaneously, connector system is a composite comparison. Ultimate loads were not normalized by declared anchorage coefficients.

### 2.2. Moisture Preparation and Experimental Groups

Eighteen full-size specimens were prepared: nine with Mitek plates (specimens 1–9) and nine with Ristek plates (10–18). Three beams per connector were initially assigned to each nominal preparation group (15%, 20%, and 40%). M1/R10 subsequently followed the nominal 40 → 20% history, whereas M9/R18 followed 15 → 20%. The direct-group comparison therefore contains 14 beams: two per connector at 15%, three at 20%, and two at 40%. The four transition beams are reported separately. The arrows describe intended histories; they do not establish measured moisture at either endpoint. Nominal preparation group, moisture at plate pressing, and moisture at bending are distinct variables. The available record does not provide separately time-stamped, verified measurements at both operations, so nominal groups must not be interpreted as an isolated assembly-moisture treatment.

Four electrical-resistance moisture TANEL HIT-3 (TANEL Elektronika i Informatyka Spółka Jawna, Gliwice, Poland) and furthermore, it was verified TANEL WRD-100 (TANEL Elektronika i Informatyka Sp. j., Gliwice, Poland) readings along each full-size member were averaged per specimen. Their timing relative to plate pressing and bending, meter model, electrode depth, species and temperature corrections, and wet-range calibration are not sufficiently documented. We use the term reported mean moisture rather than verified test-state moisture. EN 13183-2 describes an approximate working range of 7–30% [13]; values above this range cannot be accepted as quantitatively validated without an independent reference, such as oven-dry determination under EN 13183-1 [63]. The nominal 40% group was also realized differently for the two connectors (Section 3.1).

### 2.3. Full-Size Four-Point Bending

**Figure 1:**
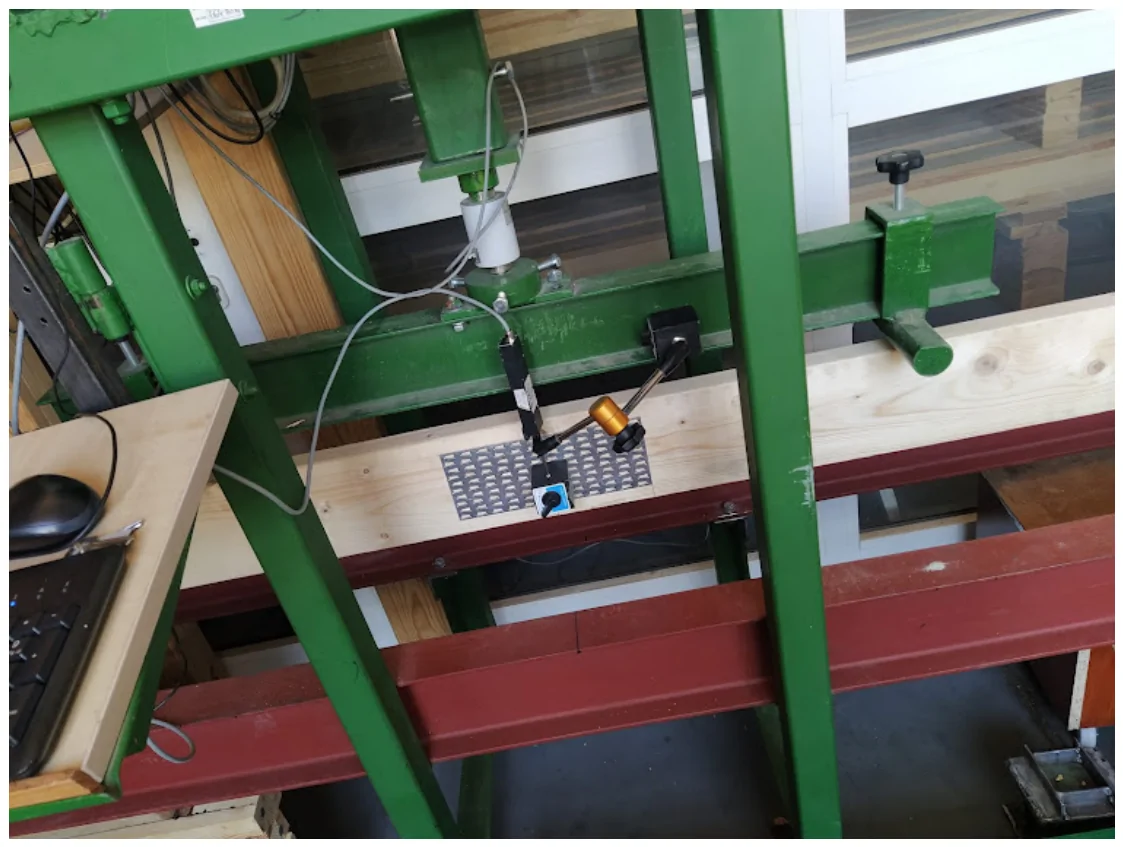
Full-size four-point bending test stand used for the metal-plate-connected specimens. Photograph from the experimental campaign.

Full-size tests were conducted on a LAB5000 testing machine (Mesdan S.p.A., Puegnago del Garda, Italy) in symmetric four-point bending. The reported support spacing was 2550 mm, crosshead speed 15 mm/min, and machine capacity 50 kN (see Figure 1). Loading continued to failure (see Table 2). Ultimate load Fmax is the primary outcome for this common reported geometry. The span-to-depth ratio was 2550/170 = 15, compared with the reference ratio of 18 in EN 408 [10]. Clauses 9.2 and 10.2 permit specified geometry adjustments for modulus tests while retaining symmetry; the ratio alone does not establish compliance or noncompliance with the complete standard. Load-point positions, displacement instrumentation, and the applicable procedure require verification. We describe the setup as an adapted four-point bending test informed by EN 408, not a fully documented EN 408 determination. Its loads must not be converted into characteristic timber strengths or compared directly with EN 338 class values. The relatively short span and the splice can increase the contribution of shear and connection deformation to global deflection.

### 2.4. Status of the Previously Tabulated Global Modulus

Beam-level means were previously reported as an apparent global flexural modulus derived from load–displacement measurements. The underlying approach, based on evaluating structural stiffness from beam deflection over defined loading intervals, is consistent with established experimental practice for assessing the global flexural response of timber assemblies.

Despite these documentation limitations, the reported values remain qualitatively consistent with the observed structural response and are therefore retained in Appendix A as a supplementary diagnostic dataset, together with the original specimen labels and all 18 beams. To avoid overinterpretation, these values are excluded from the main inferential analyses and are not used as evidence for moisture-dependent changes in stiffness. As a global deflection-derived parameter, the apparent flexural modulus reflects the combined effects of timber bending, shear deformation, connection slip and rotation, and possible seating and instrumentation influences; consequently, it should be interpreted as an indicator of overall structural response rather than a measure of local joint stiffness [21,22,23,24,47,48,49,50,51].

### 2.5. Laboratory Small-Specimen Characterization

After full-size failure, laboratory specimens nominally 20 × 20 × 300 mm were cut from the parent timber members for material characterization. For each parent beam, seven specimens were obtained from each of the two component timber pieces whenever sufficient undamaged material was available. All specimens were taken at least 300 mm from the splice region and at least 300 mm from any visible failure zone or mechanically damaged area generated during the full-scale test. Prior to testing, moisture content was verified and the measured properties were referenced to the standard moisture content of 12%.

The laboratory program included determination of bending strength (fm), proportional-limit stress, modulus of elasticity in bending (Em), bending-work index, and density calculated from specimen mass and dimensions. The specimens were conditioned to approximately 12% moisture content before four-point bending tests. The resulting material properties are therefore intended to characterize the timber material rather than the immediate moisture state present during the full-scale assembly tests.

One expected result is absent for assembly 40R3 because no suitable specimen could be obtained from the corresponding location after full-scale failure. In addition, one value in group 20R1 was excluded because the laboratory specimen exhibited mechanical damage that would have affected the measurement and was therefore rejected according to the specimen-quality criteria. These omissions are identified in the Supplementary Material.

Multiple specimens originated from each of the 18 parent assemblies. To avoid pseudoreplication, statistical summaries were based on assembly-level means rather than treating individual coupons as independent treatment replicates. Each assembly contributes one mean value, giving three parent means per connector × original preparation group. The four transition-history assemblies remain identified by their original preparation groups for descriptive purposes. Consequently, comparisons among coupon properties describe differences between the original parent groups after conditioning to approximately 12% moisture content and should not be interpreted as estimates of an immediate moisture-content effect or a connector effect on clear wood properties.

### 2.6. Statistical Analysis

Full-size inference is exploratory and conditional on independent assembly-level errors. The two-factor interaction model and an additive summary model are both reported. The additive model is a parsimonious descriptive approximation, not a model justified by proving that the interaction is absent. With *n* = 2–3 per cell, an interaction test has little ability to rule out important effect modification. The main ANOVA was checked from the reported cell counts, means, and sample standard deviations; small differences from earlier outputs reflect rounding. Table 3 gives these summary-based calculations. Partial η^2^ is retained for comparison, together with ordinary η^2^ and bias-reduced, nonpartial ω^2^ [62]. Omega-squared uses total variance as its denominator and is not directly comparable with partial η^2^. Its correction reduces small-sample bias but cannot remedy confounding or guarantee a stable population estimate.

For a term A, ω^2^ is calculated as follows, where *SS* denotes a sum of squares, *MS_E* the additive-model residual mean square, and *df_A* the degrees of freedom of that term. Partial η^2^ uses *SS_A*/(*SS_A* + *SS_E*), whereas ordinary η^2^ uses *SS_A*/*SS_total*. In Table 3, *SS_total* ≈ 50.25773 kN^2^ and *MS_E* ≈ 1.342833 kN^2^. The connector and nominal-group sums of squares are approximately 3.829406 and 32.99999 kN^2^, giving ω^2^ ≈ 0.048 and 0.587, respectively. These calculations use rounded table summaries and are not a rerun of specimen-level data.

$$\omega^{2}=\frac{{SS}_{A}-{df}_{A}\;{MS}_{E}}{{SS}_{total}+{MS}_{E}}$$

## 3. Results

### 3.1. Realized Moisture Conditions

Reported mean moisture did not reproduce the nominal preparation groups. Mitek means were 19.0%, 21.0%, and 40.45% for nominal 15%, 20%, and 40%, respectively; Ristek means were 15.5%, 19.5%, and 26.35%. Thus the nominal wet groups differ by about 14 percentage points between connector systems, in addition to uncertainty in the highest resistance readings. One nominal 15% Mitek beam had a reported mean of 21.8%. The nominal factor describes preparation labels, not matched measured exposure. Moisture and connector system are consequently entangled, and adjustment in a small regression cannot establish what either connector would have done at a common verified moisture condition (see Table 2). This is a central limitation of the comparison.

### 3.2. Ultimate Load of Full-Size MPC Members

**Figure 2:**
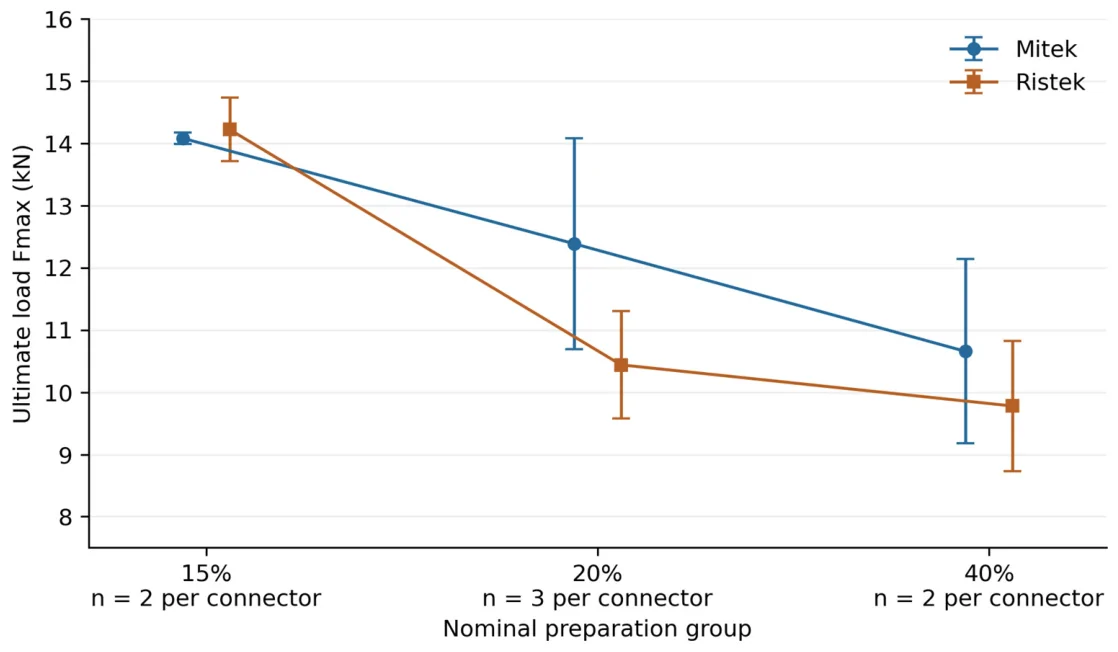
Ultimate load in the direct groups, summarized from Table 2. Markers show connector-specific means, bars show ±1 sample SD, and n gives the number of assemblies in each cell. Lines connect means only. The horizontal groups are nominal preparation labels, not separately verified moisture contents at assembly or testing.

Across both connector systems, mean ultimate load was 14.152 kN in the nominal 15% group, 11.413 kN at 20%, and 10.217 kN at 40% (pooled *n* = 4, 6, and 4). The latter means were 19.4% and 27.8% lower than the first. These are between-beam differences across combined preparation and test histories, not losses measured within the same beams. Figure 2 shows the connector-specific summaries. Neither the percentage differences nor the low observed scatter of the four nominally driest beams establishes a general moisture reduction factor or manufacturing repeatability.

The summary-based interaction test gave F(2,8) ≈ 0.985, *p* ≈ 0.415. This does not establish parallel connector responses. In the additive model, the nominal-group association was F(2,10) ≈ 12.29, *p* ≈ 0.002, with partial η^2^ ≈ 0.711, ordinary η^2^ ≈ 0.657, and bias-reduced ω^2^ ≈ 0.587. The connector term gave F(1,10) ≈ 2.85, *p* ≈ 0.122, partial η^2^ ≈ 0.222, ordinary η^2^ ≈ 0.076, and ω^2^ ≈ 0.048. Its nonsignificance cannot establish equivalence or exclude a practically important system difference. All estimates are imprecise and conditional on the additive model and independence assumption. The earlier rank-based summary was H(2) = 8.381 with asymptotic *p* = 0.015. Earlier Holm-adjusted exact pairwise *p*-values were 0.0286, 0.0571, and 0.476 for 15 versus 20, 15 versus 40, and 20 versus 40, respectively. Pooled group sizes of 4, 6, and 4 give very limited rank-test resolution; these comparisons are not independent confirmation of a causal moisture effect (Table 3).

**Figure 3:**
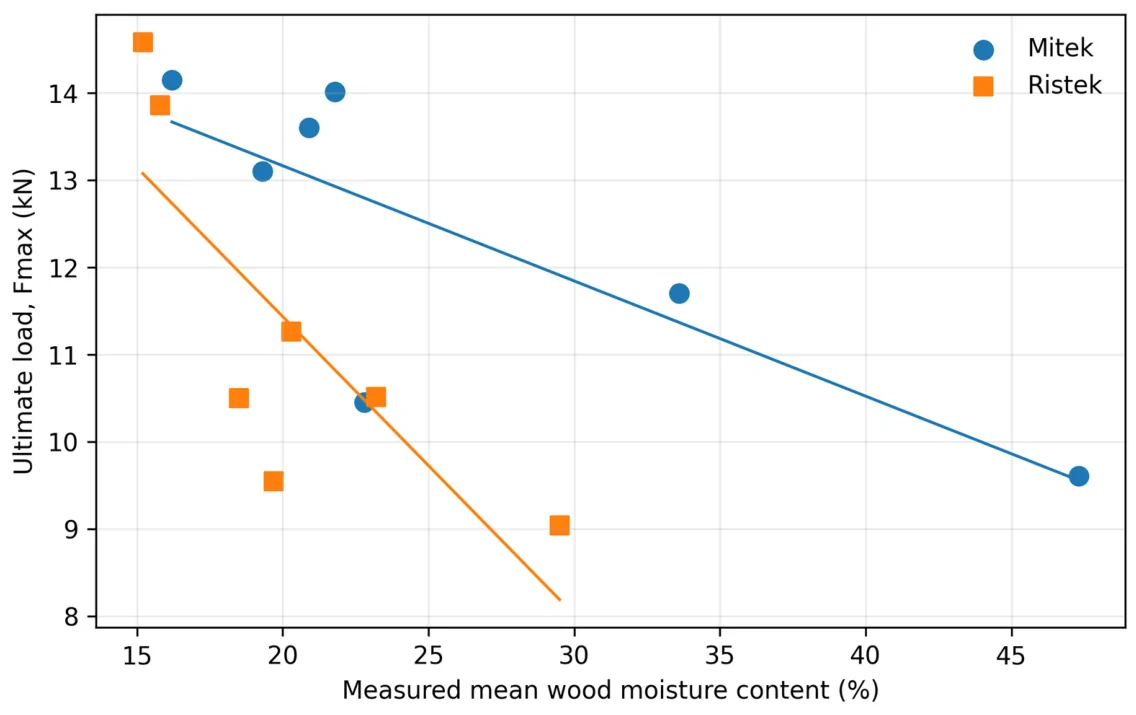
Original plot of reported mean moisture readings and ultimate load in the 14 direct specimens. Lines are descriptive least-squares visual aids only; they are not confidence limits, validated dose–response models, or tests of connector superiority. Readings above approximately 30% lack documented independent validation. The plotted moisture values are not separately verified at both pressing and testing.

The previously reported Spearman association between mean moisture reading and ultimate load was ρ = −0.591 (*p* = 0.026). Figure 3 retains the original observations and descriptive connector-specific lines. The association is a summary of this small sample, with uncertain wet-range measurements and non-comparable moisture distributions; it is not a calibrated dose–response relationship. In particular, a straight line across approximately 15–50% cannot represent a verified common physical slope below and above fiber saturation. No adjusted HC3 coefficient is interpreted as a moisture or connector effect.

### 3.3. Single-Specimen Moisture-History Cases

The four transition-history specimens cannot support inferential comparisons because each connector × history condition contains only one beam. The 40 → 20 specimens failed at 10.836 kN (Mitek) and 9.236 kN (Ristek), approximately 12.5% and 11.5% below their respective direct-20% connector means. The 15 → 20 specimens gave 10.320 kN (Mitek) and 13.726 kN (Ristek), corresponding to −16.7% and +31.5% relative to the respective direct-20% means. The opposite directions of the two 15 → 20 cases show why these observations should not be used to claim a deterministic history effect. They are retained as case evidence motivating replicated cycling experiments rather than folded into the primary factorial test.

### 3.4. Status of the Global Modulus Diagnostic

**Figure A1:**
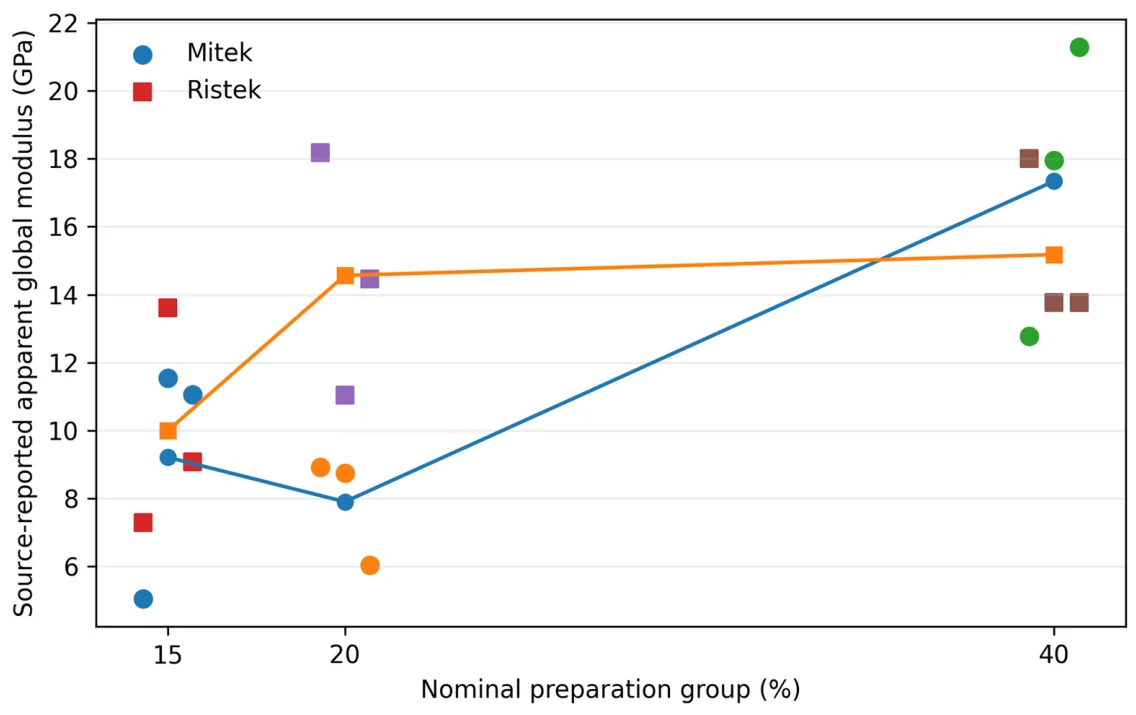
Original global-modulus plot retained as a confounded, unvalidated diagnostic. All 18 assemblies are grouped by initial preparation labels, including transition cases. The apparent wet-group increase cannot be attributed to a beneficial moisture effect, and no inferential *p*-value is attached to this plot.

The previously reported apparent global moduli show a nonmonotonic pattern, including higher wet-group means. This does not demonstrate a moisture-driven increase in either wood stiffness or joint stiffness. Between-beam timber differences, transition histories, and incompletely documented calculation and displacement measurements offer competing explanations that cannot be separated. Table A1 and Figure A1 preserve these summaries as a confounded, unvalidated diagnostic. No inferential test of a moisture-related stiffness effect is reported.

### 3.5. Nested Laboratory Specimens and Density

**Figure 4:**
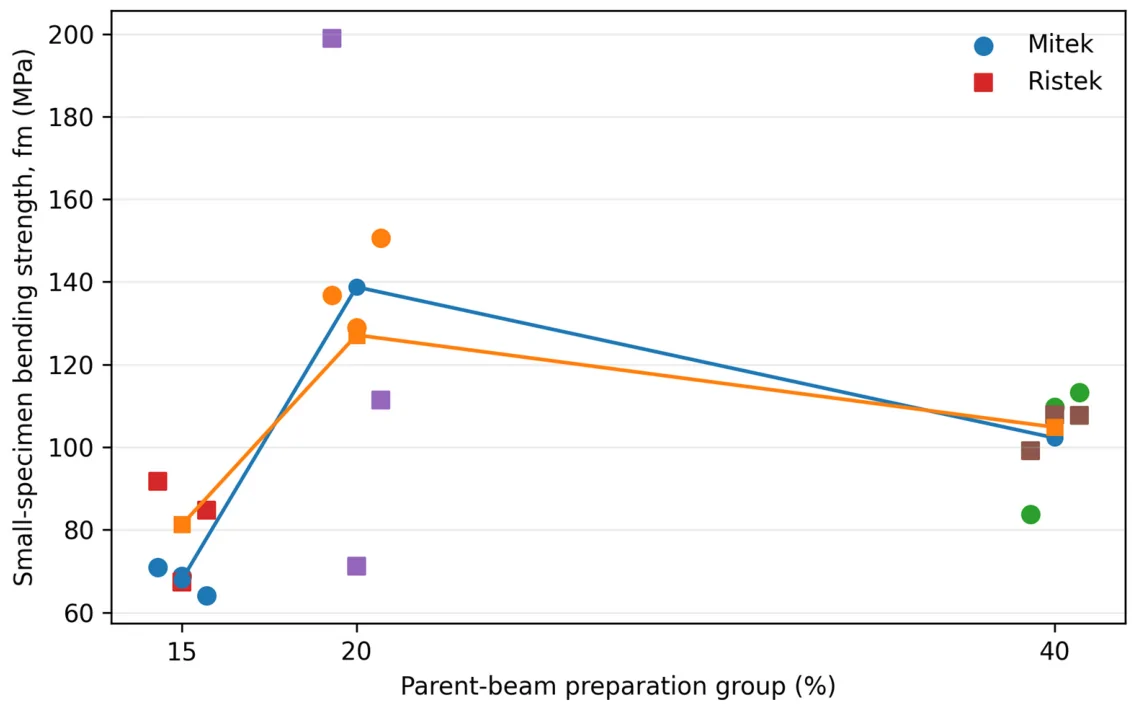
Laboratory bending strength summarized at the parent-assembly level. Each point is a parent mean, not an independent coupon. The original preparation labels include transition-history beams; connecting lines are descriptive, and the coupons were subsequently conditioned to approximately 12%.

Table 4 summarizes one laboratory mean per parent assembly, with three parent means in each connector × original preparation cell. The 15/20/40 labels describe initial parent groups, including the four transition cases; they do not describe moisture at coupon testing. The protocol specified conditioning to approximately 12%, but coupon-level final moisture and sampling-position records are not available here. Figure 4 therefore depicts between-parent variation after their differing histories, not a moisture–strength response curve. The original coupon and parent-beam tables are needed to verify individual observations, including the previously noted missing observation in 40R3.

The earlier parent-mean analysis reported interaction *p*-values of 0.753 for fm, 0.622 for proportional-limit stress, and 0.481 for Em. These small-sample tests do not establish that interactions are absent. Reported additive parent-group statistics were F(2,14) = 7.045 (*p* = 0.0076) for fm, 9.094 (*p* = 0.0029) for proportional-limit stress, and 12.943 (*p* = 0.00066) for Em (Table 5). Connector *p*-values were 0.912, 0.870, and 0.580. These are exploratory descriptions of material-group heterogeneity, conditional on assembly-level independence, not treatment effects. Multiple outcomes were examined, and the displayed omnibus *p*-values have not been adjusted across outcomes. The bending-work index is excluded from testing because its physical definition is unresolved; unverifiable permutation *p*-values are also omitted.

**Figure 5:**
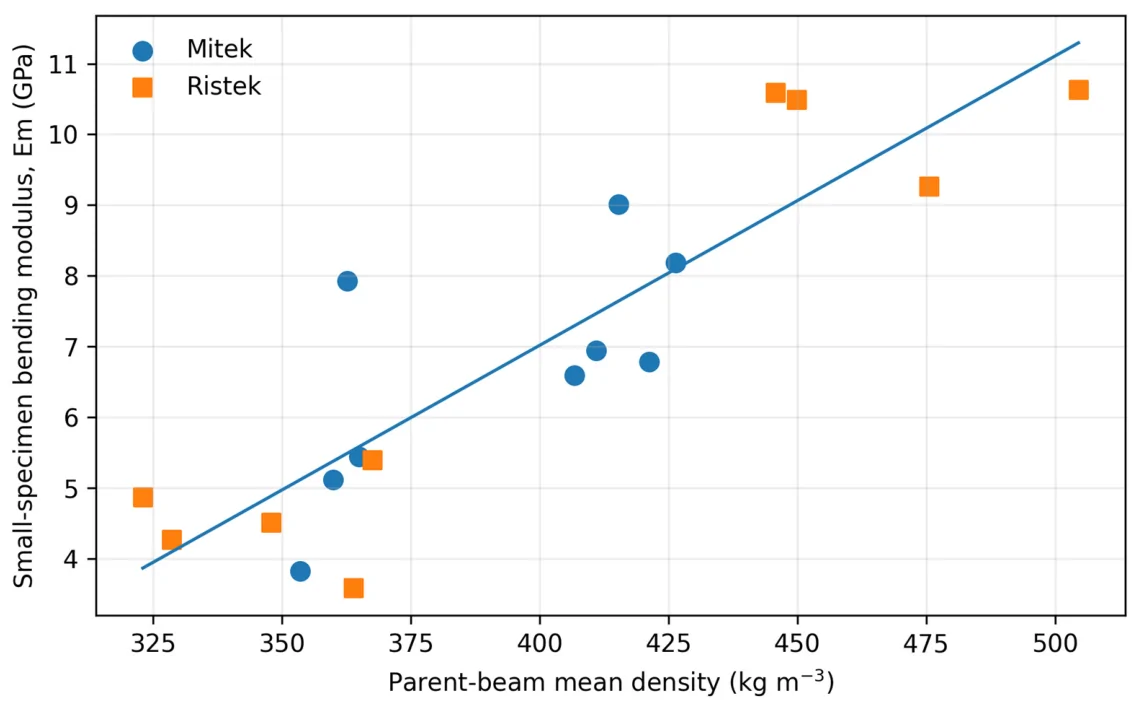
Previously reported parent-assembly mean density and laboratory bending modulus. The overall least-squares line is a descriptive visual aid. The reported rank association (ρ = 0.874) is exploratory and depends on the unverified independence of parent assemblies.

The previously reported Holm-adjusted exact rank comparisons separated the 15% parent group from both other groups for fm (*p* = 0.026 for each comparison) and proportional-limit stress (*p* = 0.0087 and 0.0065), whereas 20% versus 40% did not reach the conventional threshold. For Em, only 15% versus 40% did so (*p* = 0.0065). These sparse comparisons remain sensitive to parent selection, sampling after failure, and unknown batches. The reported parent-mean density association was stronger for Em (Spearman ρ = 0.874, *p* = 2.1 × 10^−6^) than for fm (ρ = 0.003, *p* = 0.990). This pattern is compatible with material heterogeneity, but does not identify its origin or eliminate the confounding in the full-size comparison (see Figure 5).

### 3.6. Failure Mechanisms

Visual examples records and narrative descriptions indicate that several damage mechanisms occurred in the tested assemblies and that the observed behavior cannot be reduced to a single “plate failure” category. Documented damage included timber splitting and cracking adjacent to the splice, local crushing of wood fibers, plate displacement, partial tooth withdrawal, plate bending, and, in some cases, plate fracture.

For the Ristek specimens, the wet-condition group exhibited timber cracking adjacent to the joint. The 20% preparation group showed failures located primarily in the joint region beneath the connector plate, while the 15% group included cases of nail-plate fracture in the lower chord. For the Mitek specimens, the wet-condition group exhibited compression-zone crushing and timber cracking near the joint. The 20% group showed plate disturbance, displacement, partial withdrawal, and local wood damage, whereas the 15% group included joint-zone cracking and plate deformation.

These observations are based on available photographs and test records. However, the documentation does not contain a complete specimen-by-specimen classification of damage mechanisms at either first failure or final collapse. The observations reported in Table 6 therefore distinguish documented damage features from unavailable frequency data, and the numbers of specimens in each group should not be interpreted as counts of a particular failure mode.

**Figure 6:**
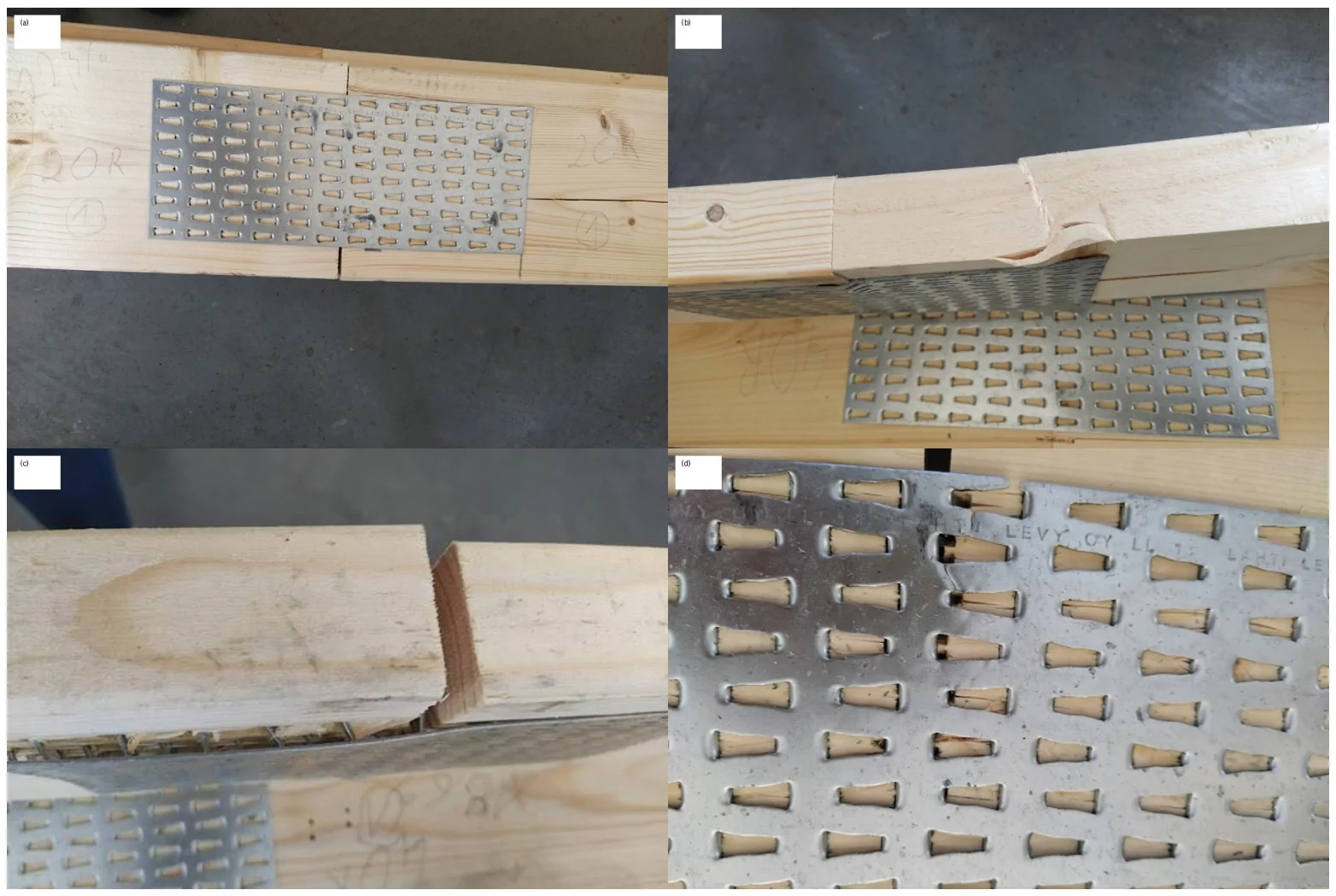
Representative photographs retained from the industrial campaign: (**a**) Ristek, nominal 20%; (**b**) Mitek, nominal 40%; (**c**) Mitek, nominal 20%; (**d**) Ristek, nominal 15%. The images do not provide group frequencies or establish the sequence of failure mechanisms.

The representative photographs presented in Figure 6 illustrate typical damage appearances only. Because multiple damage mechanisms may coexist within a single assembly, assignment of a unique dominant failure mode would require a predefined classification rule and a complete specimen-level failure log.

## 4. Discussion

### 4.1. Moisture Preparation and Full-Size Capacity

The main observation is a lower mean full-size ultimate load in the nominally wetter preparation groups. Its direction is compatible with established moisture sensitivity of timber below the fiber-saturation region [14,15] and of plate–wood interfaces [16,17,18,19,20,21,22,23]. Compatibility with those mechanisms is not their identification in this experiment. Moisture during pressing may affect tooth embedment and seating, whereas moisture during bending changes the timber response at the time of loading. Without separate verified measurements and independently crossed treatments, both contributions are combined with between-specimen timber differences.

The approximately 28% difference between the nominal 15% and 40% pooled means is therefore a sample description. It is not directly comparable with within-specimen deterioration or accelerated-soaking losses in earlier MPC research [16,17,18]. The two wet connector groups also had different reported moisture, and the highest readings lack independent validation. Although the bias-reduced group estimate (ω^2^ ≈ 0.587) is smaller than the ordinary η^2^ estimate (≈0.657), neither removes selection bias or the instability of *n* = 2–3 cells. The design supports a hypothesis for controlled testing, not a quantified causal penalty for assembling wet timber.

### 4.2. Connector System, Geometry, and Semi-Rigid Response

The connector comparison is inconclusive. A partial η^2^ of approximately 0.222 together with *p* ≈ 0.122 is compatible with a material system difference that this study cannot estimate reliably; it must not be summarized as no connector effect. Moreover, plate dimensions, thickness, steel grade, and tooth configuration differ together, while realized moisture and possibly timber batches also differ. Existing MPC studies show that geometry, orientation, gap, and combined loading can influence resistance [19,22,32,33,34,35,36,37,38,39,40,41]. The present comparison cannot assign the observed differences to a manufacturer or an individual connector characteristic.

Removing the HC3 connector coefficient avoids implying that conditioning on one uncertain moisture reading produces a comparison at matched physical conditions. Such a regression would require an adequate common response form, sufficient exposure overlap, reliable moisture measurement, independent errors, and no omitted timber or batch factors linked to connector assignment. None is established here. A more informative comparison would balance verified moisture states, document allocation and batches, and use either matched geometry or a prespecified mechanical normalization of anchorage area.

The global-modulus diagnostic likewise cannot identify a beneficial effect of wetting. Semi-rigid analyses show that joint rotation and slip contribute to beam deflection [24,47,48,49,50,51,52,53,54], but the present record does not quantify those components. The counterintuitive pattern could arise from material variation, moisture history, seating, or measurement and calculation choices. The appropriate response is to withhold stiffness inference until the load–displacement records are audited. Direct local slip or rotation measurements would be required for a connection-stiffness claim [21,23,51,60,64,65].

### 4.3. Parent-Material Variability and the Small-Specimen Tests

Averaging coupons within each parent assembly addresses one level of pseudoreplication. It does not correct unknown common-stock or production-batch dependence among full-size assemblies, nor does it resolve unequal sampling of the two timber pieces in each splice. Because coupons were cut after failure and then conditioned, their group differences may combine original material variability, prior history, damage, and selection of surviving regions. They should not be used as an independent demonstration of a moisture-treatment effect.

The reported density–Em association (ρ = 0.874) is consistent with a contribution from material variability. The absence of a corresponding density–fm association in the 18 parent assembly means does not establish that density is generally unrelated to strength. Local anatomy, grain deviation, defects, and the sampling procedure may contribute [1,3,15]. The unusually high 20R1 coupon response relative to 20R2 and 20R3 further warrants examination of the underlying specimen and sampling records. Parent means preserve the assembly as the analysis unit, but cannot reconstruct a randomized experiment.

### 4.4. Relation to the Independent Cyclic-Conditioning Study

The independent Materials study by Wieruszewski et al. [2] provides a useful methodological contrast. In that experiment, 15 MPC specimens were repeatedly measured through wetting–drying stages; the mean apparent modulus decreased from 1.39 to 1.22 GPa (about 12%), but the Friedman repeated-measures test was not significant (*p* = 0.127), and the authors explicitly avoided attributing the trend solely to moisture because the same beams were repeatedly loaded and no unexposed MPC control group was included. The mean apparent bending strength of the final MPC assemblies was 16.92 MPa, compared with 34.14 MPa for structurally different continuous references, and failure was dominated by plate slip and partial tooth withdrawal [2].

That repeated-measurement study and the present between-beam comparison address different questions. Neither justifies attributing every moisture-associated difference to one interface mechanism. Here, the specific obstacles are unseparated assembly and test-state moisture, unmatched realized exposure, incomplete allocation records, and very small cells. A future program should cross assembly and test moisture independently, retain matched MPC controls, and allocate destructive tests to prespecified histories. Those features would help distinguish the mechanisms considered in the environmental, cyclic, fatigue, and creep literature [16,17,18,25,26,27,28,29,30,31].

### 4.5. Engineering Implications and Limitations

For fabrication practice, the established MPC literature supports control and documentation of timber moisture [14,15,16,17,18]. The present exploratory comparison reinforces the need to record actual conditions, but does not identify an optimum pressing moisture or establish process repeatability. The reported loads belong to these specimens and this adapted geometry. They cannot replace resistance verification, service-class provisions, or product-control requirements [5,6,7,8,9], and the sample is inadequate for estimating characteristic resistance.

The principal limitations affect identifiability, not just statistical power: assembly and test-state moisture cannot be separated; wet-range readings are unvalidated; connector system and reported moisture are associated; and randomization and batch allocation are undocumented. The *n* = 2–3 cells leaves important connector differences and interactions unresolved. Additional limitations are inconsistent component-length reporting, incomplete displacement and modulus provenance, one beam per transition-history cell, no local slip/rotation measurement, post-failure coupon sampling without a sampling map, and no complete failure-mode census. Statistical adjustment cannot reconstruct missing treatment contrasts, recover unknown blocks, or validate a measurement outside its documented range.

A follow-up experiment should use documented randomization within timber batches, blocking or matching by density, grade, and relevant defects. Assembly moisture and test moisture should be crossed as separate factors, including wet-assembled beams subsequently tested at a common verified moisture alongside appropriate dry-assembled controls. Wet-range measurements should be checked against an oven-dry reference. Local slip/rotation, global deflection, loading-system compliance, and failure onset should be recorded. Coupon locations should be mapped to both component pieces and damaged regions. The number of independent beams should be chosen by an a priori precision or power analysis for an engineering-relevant difference, rather than from the inflated effect estimate of this small study. If batches define the treatment units, replication must also occur at that level.

## 5. Conclusions

1. In 14 directly conditioned assemblies, pooled mean ultimate load decreased across nominal preparation groups from 14.15 to 11.41 and 10.22 kN. The additive group association (F(2,10) ≈ 12.29, *p* ≈ 0.002; bias-reduced, nonpartial ω^2^ ≈ 0.587) is exploratory, uncertain, and conditional on the model assumptions.
2. These differences cannot be attributed specifically to assembly moisture. Moisture at pressing and testing is not separately verified, the nominal wet groups differ in reported moisture by about 14 percentage points between connectors, and the highest resistance readings lack independent validation.
3. The connector comparison is inconclusive. Its *p* ≈ 0.122 and partial η^2^ ≈ 0.222 neither establish equivalence nor exclude a practically relevant difference. Sparse cells, product differences, and possible timber-batch confounding also prevent reliable interaction or manufacturer-specific conclusions.
4. The previously tabulated global modulus is an unvalidated, confounded diagnostic, not evidence that wetting increases stiffness. No moisture-related stiffness claim is made. Local joint stiffness requires direct, documented connection measurements.
5. Parent-assembly aggregation avoids counting coupons as independent treatment replicates, but cannot remove unknown batch dependence or post-failure sampling bias. The practical contribution is a documented, exploratory association and a clearer design for follow-up testing, not a moisture reduction factor, an optimum assembly condition, or a connector ranking.

## Figures and Tables

**Table 1:** Reported connector dimensions and material descriptions. No tabulated property was used to normalize ultimate load.

Characteristic	Mitek T150	Ristek LL13
Nominal plate size	124 × 245 mm	120 × 300 mm
Steel grade	S250GD + Z275	S350GD + Z275-M-A
Sheet thickness	1.5 mm	1.3 mm

**Table 2:** Direct full-size groups with reported moisture readings and ultimate load (mean ± sample SD). Moisture readings above the validated meter range remain unverified.

Connector	Nominal Group	*n*	Reported Mean Moisture (%)	Fmax (kN)	CV of Fmax (%)
Mitek	15	2	19.0 ± 4.0	14.082 ± 0.095	0.67
Mitek	20	3	21.0 ± 1.8	12.387 ± 1.693	13.66
Mitek	40	2	40.45 ± 9.69	10.656 ± 1.481	13.90
Ristek	15	2	15.5 ± 0.4	14.221 ± 0.511	3.59
Ristek	20	3	19.5 ± 0.9	10.439 ± 0.863	8.27
Ristek	40	2	26.35 ± 4.45	9.778 ± 1.044	10.67

**Table 3:** Exploratory full-size ANOVA checked from the rounded summaries in Table 2. All numerical estimates are approximate.

Term	df	F	*p*	Partial η^2^	η^2^	ω^2^
Connector × group	2, 8	0.985	0.415	0.198	—	—
Connector additive	1, 10	2.85	0.122	0.222	0.076	0.048
Nominal group additive	2, 10	12.29	0.002	0.711	0.657	0.587

Note: Ordinary η^2^ and ω^2^ use total variance; partial η^2^ excludes the other fitted term from its denominator. The additive residual has 10 degrees of freedom; the interaction model has 8. The interaction is retained as a sensitivity description, and nonsignificance does not establish its absence. Omega-squared is reported for the additive summaries only. Rank and correlation outputs discussed in the text were not recalculated from these aggregate data.

**Table 4:** Previously reported parent-assembly means of laboratory small-specimen properties (mean ± SD across three parent assemblies per original preparation cell). Transition histories remain in the original groups.

System	Initial Group	fm (MPa)	Proportional Limit (MPa)	Em (GPa)	Bending-Work Index *
Mitek	15	67.95 ± 3.50	21.28 ± 5.68	8.37 ± 0.56	8.24 ± 2.20
Mitek	20	138.77 ± 10.99	65.08 ± 10.23	6.77 ± 0.18	124.59 ± 32.46
Mitek	40	102.23 ± 16.10	44.37 ± 4.55	4.79 ± 0.85	79.87 ± 6.06
Ristek	15	81.26 ± 12.45	30.87 ± 5.85	10.11 ± 0.74	12.17 ± 2.34
Ristek	20	127.14 ± 65.37	57.58 ± 33.55	6.53 ± 3.64	100.32 ± 56.78
Ristek	40	104.83 ± 4.97	45.67 ± 3.06	4.54 ± 0.30	93.46 ± 17.43

* The bending-work quantity retains its earlier numerical scale. Its dimensional definition is unverified; it is a descriptive index and is excluded from inferential and cross-study physical comparisons.

**Table 5:** Previously reported exploratory tests on laboratory parent means. These are unadjusted omnibus *p*-values, not evidence of an isolated moisture or connector effect.

Outcome	Connector *p*	Parent Group F(2,14)	Parent Group *p*	Partial η^2^ Group
fm	0.912	7.045	0.0076	0.502
Proportional limit	0.870	9.094	0.0029	0.565
Em	0.580	12.943	0.00066	0.649

**Table 6:** Qualitative failure descriptions by direct group. A complete specimen-level mode census is not available.

Connector and Group	Direct n	Mechanisms Described in the Earlier Narrative	Mode Count
Mitek 15%	2	Joint-zone cracking; plate bending	ND
Mitek 20%	3	Plate disturbance/displacement; partial pullout; local wood damage	ND
Mitek 40%	2	Compression-zone crushing or wood cracking near the joint	ND
Ristek 15%	2	Plate fracture	ND
Ristek 20%	3	Failure in the joint zone beneath the plate	ND
Ristek 40%	2	Wood cracking adjacent to the joint	ND

ND = not documented, not zero. The mechanisms are group-level narrative descriptions; a one-to-one link to all direct specimens has not been established. Frequencies and percentages cannot be calculated from these descriptions. M1/R10 and M9/R18 are transition cases and are excluded from the direct-group denominators. Mixed mechanisms require an explicit classification rule.

## Data Availability

Group summaries and representative figures are included in this article. The complete specimen-level tables, primary test records, load–displacement files, and executable analysis are required to reproduce all reported outcomes; their archive location and access conditions remain to be specified.

## Supplementary Materials

The following supporting information can be downloaded at: https://www.mdpi.com/article/10.3390/polym18192393/s1, Reproducible specimen-level verification requires the original coupon tables (S1–S6), parent-assembly means and identifiers (S7), full-size observations and moisture histories (S8), and executable statistical analysis with diagnostics (S9). Their contents are not reproduced in this manuscript. Appendix A and Appendix B describe the modulus and computational limitations; they do not replace those data.

## Abbreviations

MPC	metal-plate-connected
C24	structural softwood strength class
Fmax	ultimate load
fm	bending strength
Em	bending modulus
DIC	digital image correlation
HC3	heteroscedasticity-consistent covariance estimator
CV	coefficient of variation

## Appendix A. Confounded Global Modulus Diagnostic

The following summaries preserve the previously tabulated values for traceability. They are not validated estimates of intrinsic timber modulus or local joint stiffness. The original grouping contains all 18 beams, including M1/R10 (40 → 20%) and M9/R18 (15 → 20%). The 15/20/40 headings refer only to original preparation groups, with three beams per connector and group.

**Table A1:** Previously tabulated apparent global modulus (GPa; mean ± SD). Original grouping only; measurement timing and calculation provenance are unresolved.

Connector	15% (GPa)	20% (GPa)	40% (GPa)
Mitek	9.22 ± 3.62	7.91 ± 1.62	17.34 ± 4.29
Ristek	9.99 ± 3.26	14.57 ± 3.57	15.18 ± 2.45

## Appendix B. Reproducibility Requirements for the Permutation Analysis

For the nominal-group test in an additive full-size model, the reduced model contains an intercept and connector system. For the connector test, it contains an intercept and nominal group. For an interaction test, the reduced model contains both main effects and the full model adds their interaction. Fit the relevant reduced model, permute its assembly-level residuals under a justified exchangeability scheme, add them to the reduced-model fitted values, and refit the full and reduced models to each constructed response. Compare the resulting partial F statistics with the observed statistic. With B random permutations, calculate the Monte Carlo *p*-value as (b + 1)/(B + 1), where b counts statistics at least as large as the observed value. Record B, the random-number generator and seed, the model matrices, and software versions.

Freedman–Lane handles nuisance terms through the reduced model. It differs from Kennedy residualization and ter Braak full-model residual permutation; comparative simulations provide a rationale for considering it, not a guarantee for this dataset [63]. Exchangeability must be justified for the actual allocation and error structure. Unknown stock or production blocks may invalidate unrestricted permutations, and very small or completely confounded blocks may leave no informative permutation test. Coupon residuals would be permuted only after aggregation to parent means, with any documented higher-level blocks respected. The procedure cannot fully remove the moisture–connector confounding or compensate for unvalidated moisture readings.
