# Supplementary material for: Moisture Preparation and Bending Performance of Metal Plate Connected C24 Spruce Members in an Exploratory Comparison of Two Connector Systems
Source: Polymers (Basel). 2026 Sep 30;18(19):2393. doi: 10.3390/polym18192393 (PMC13666639; doi:10.3390/polym18192393)
Supplement: Supplementary file 1 [file polymers-18-02393-s001.zip › polymers-4558664-supplementary.pdf]

Supplementary Materials

Supplementary Materials: Moisture Condition at Assembly and Bending Performance of Metal-Plate-Connected C24 Spruce Members

Marek Wieruszewski, Weronika Magdziak, Arkadiusz Gronowski and Agnieszka Katarzyna Wdowiak-Postulak *

* Correspondence: awdowiak@tu.kielce.pl

S1. Source Table 17

Table S1. Original Table 17: four-point bending results for laboratory specimens from Ristek-connected parent beams, 15% preparation group.

Test / group	fm (N/mm²)	Proportional-limit stress (MPa)	Em (MPa)	Bending work (source: J/mm³)
15 R1	15 R1	15 R1	15 R1	15 R1
1	93,6	41,36	10456	19,17
2	91,7	37,95	10152	16,67
3	90,8	38,84	9730	12,26
4	83,5	33,82	10551	12,82
5	93,4	36,71	11684	13,48
6	91,7	36,58	10568	14,92
7	97,3	37,19	10955	14,69
Stan. deviation	4,2	2,31	617	2,41
Average	91,7	37,49	10585	14,86
15 R2	15 R2	15 R2	15 R2	15 R2
1	77,2	32,46	9748	12,64
2	84,4	37,35	9777	17,45
3	55,6	8,07	9242	1,048
4	50,8	7,84	8622	0,98
5	70,1	41,81	8586	23,54
6	77,1	30,04	10493	10,18
7	55,6	24,89	8342	8,62
Stan. deviation	13,2	13,47	789	8,23
Average	67,3	26,07	9259	10,64
15 R3	15 R3	15 R3	15 R3	15 R3

Test / group	fm (N/mm²)	Proportional-limit stress (MPa)	Em (MPa)	Bending work (source: J/mm³)
1	87,1	15,98	10635	3,074
2	92,9	40,30	11366	16,92
3	83,2	10,21	11226	1,385
4	83,3	32,27	10991	11,12
5	77,0	37,15	9031	17,53
6	85,1	30,37	10600	10,51
7	84,8	36,98	9588	16,48
Stan. deviation	4,77	11,5	869	6,62
Average	84,77	29,04	10491	11,00

S2. Source Table 18

Table S2. Original Table 18: four-point bending results for laboratory specimens from Ristek-connected parent beams, 20% preparation group.

Test / group	fm (N/mm²)	Proportional-limit stress (MPa)	Em (MPa)	Bending work (source: J/mm³)
20R1	20R1	20R1	20R1	20R1
1	194,4	79,5	9356,5	126,9
2	162,8	106,9	9241,9	232,0
3	206,1	92,2	11139,1	141,0
4	233,4	100,2	11421,9	163,0
5	182,7	84,0	9695,8	135,3
6	214,2	112,8	12914,5	195,1
Stan. deviation	24,8	13,0	1450,9	40,8
Average	198,9	95,9	10628,3	165,6
20 R2	20 R2	20 R2	20 R2	20 R2
1	101,9	50,8	4761,6	100,5
2	53,4	21,8	2958,6	30,02
3	26,8	16,9	2874,7	15,6
4	78,2	33,5	3034,2	69,1
5	26,5	15,7	2759,7	14,9
6	100,4	45,9	4306,8	91,7
7	110,9	49,4	4351,4	104,6

Test / group	fm (N/mm²)	Proportional-limit stress (MPa)	Em (MPa)	Bending work (source: J/mm³)
Stan. deviation	35,84	15,45	853,8	40,02
Average	71,15	33,41	3578,1	60,93
20 R3	20 R3	20 R3	20 R3	20 R3
1	118,9	69,1	5504,4	158,5
2	114,8	52,5	5442,4	93,8
3	108	52,0	5745,4	87,0
4	149,3	60,5	6301,4	107,0
5	126,6	16,8	5268,3	11,5
6	67,5	25,2	4083,1	29,3
7	82,4	18,1	5367,0	11,8
8	123,5	53,3	5388,9	96,6
Stan. deviation	25,81	20,31	620,6	52,20
Average	111,38	43,43	5387,6	74,44

S3. Source Table 19

Table S3. Original Table 19: four-point bending results for laboratory specimens from Ristek-connected parent beams, 40% preparation group.

Test / group	fm (N/mm²)	Proportional-limit stress (MPa)	Em (MPa)	Bending work (source: J/mm³)
40R1	40R1	40R1	40R1	40R1
1	103,7	63,3	4425,5	165,8
2	85,2	56,1	3988,3	147,1
3	102,3	44,22	4695,3	77,8
4	126,9	49,81	4855,3	94,7
5	85,6	19,14	3442,6	20,92
6	91,8	48,68	4306,1	102,4
7	98,1	62,00	4139,3	176,7
Stan. deviation	14,34	14,94	470,77	55,03
Average	99,09	49,04	4264,63	112,20
40R2	40R2	40R2	40R2	40R2
1	105,8	53,9	3789,1	105,7
2	112,3	52,29	4587,8	109,6

Test / group	fm (N/mm²)	Proportional-limit stress (MPa)	Em (MPa)	Bending work (source: J/mm³)
3	126,7	2,93	5735,5	0,3
4	117	68,08	5136,7	172,1
5	82,4	36,92	2886,4	94,6
6	107,5	44,93	4801,0	77,5
7	102,7	42,71	4602,6	72,9
Stan. deviation	13,77	20,33	926,42	51,46
Average	107,77	43,11	4505,59	90,39
40 R3	40 R3	40 R3	40 R3	40 R3
1	111,7	42,77	4737,6	72,7
2	-	-	-	-
3	94,2	38,17	4294,3	62,9
4	110,9	44,46	5188,1	69,9
5	108,5	44,9	5284,8	71,6
6	98,9	45,41	4558,7	85,3
7	121,6	53,5	5097,8	104,4
Stan. deviation	8,94	4,55	358,34	13,62
Average	107,63	44,87	4860,22	77,80

S4. Source Table 20

Table S4. Original Table 20: four-point bending results for laboratory specimens from Mitek-connected parent beams, 15% preparation group.

Test / group	fm (N/mm²)	Proportional-limit stress (MPa)	Em (MPa)	Bending work (source: J/mm³)
15M1	15M1	15M1	15M1	15M1
1	59,6	12,98	5355	3,967
2	86,6	44,22	8294	27,17
3	85,6	33,94	11515	11,66
4	46,01	18,48	9587	4,021
5	77,4	30,43	9734	11,12
6	61,6	26,4	9474	8,54
7	79,7	2,516	9098	0,1155
Stan. deviation	14,22	12,92	1741,38	8,19

Test / group	fm (N/mm²)	Proportional-limit stress (MPa)	Em (MPa)	Bending work (source: J/mm³)
Average	70,93	24,14	9008,14	9,51
15 M2	15 M2	15 M2	15 M2	15 M2
1	70,0	0,60	8203	0,02
2	66,1	25,76	8424	9,26
3	65,9	1,08	7102	0,04
4	70,0	28,12	7869	11,72
5	72,4	30,87	8580	13,06
6	68,6	1,98	7387	0,10
Stan. deviation	2,29	13,60	536,22	5,76
Average	68,83	14,73	7927,50	5,70
15 M3	15 M3	15 M3	15 M3	15 M3
1	43,7	21,99	8031	6,92
2	50,3	17,50	7540	5,11
3	65,0	28,53	5605	16,74
4	62,3	28,50	7798	12,03
5	78,1	24,22	9415	7,40
6	71,9	26,70	8718	9,70
7	77,3	27,33	10194	8,58
Stan. deviation	12,20	3,76	1366,90	3,59
Average	64,09	24,97	8185,86	9,50

S5. Source Table 21

Table S5. Original Table 21: four-point bending results for laboratory specimens from Mitek-connected parent beams, 20% preparation group.

Test / group	fm (N/mm²)	Proportional-limit stress (MPa)	Em (MPa)	Bending work (source: J/mm³)
20 M1	20 M1	20 M1	20 M1	20 M1
1	161,9	63,5	8282,3	91,5
2	121,2	6,7	6258,3	1,5
3	146,1	66,7	6874,5	119,1
4	135,1	76,9	6097,1	179,5
5	99,5	48,0	6566,1	64,9

Test / group	fm (N/mm²)	Proportional-limit stress (MPa)	Em (MPa)	Bending work (source: J/mm³)
6	147,4	59,1	6967,4	93,1
7	146,2	57,4	7556,6	81,0
Stan. deviation	19,10	20,99	707,30	49,87
Average	136,77	54,05	6943,19	90,09
20 M2	20 M2	20 M2	20 M2	20 M2
1	99,2	63,8	4549,3	163,4
2	146,8	75,7	6896,3	155,1
3	112,0	70,4	6982,0	130,7
4	176,4	72,4	8117,3	120,7
5	106,6	58,5	5771,2	109,6
6	132,9	60,7	7214,7	95,4
Stan. deviation	26,64	6,31	1140,56	23,95
Average	128,98	66,92	6588,47	129,15
20 M3	20 M3	20 M3	20 M3	20 M3
1	165,0	78,8	7021,4	164,8
2	149,4	83,4	6013,8	213,1
3	125,2	56,1	4629,6	128,4
4	159,4	69,7	7022,3	129,2
5	179,5	97,2	8193,1	215,5
6	88,8	52,7	6363,1	80,4
7	186,6	82,0	8228,3	150,4
Stan. deviation	31,35	14,67	1170,87	44,86
Average	150,56	74,27	6781,66	154,54

S6. Source Table 22

Table S6. Original Table 22: four-point bending results for laboratory specimens from Mitek-connected parent beams, 40% preparation group.

Test / group	fm (N/mm²)	Proportional-limit stress (MPa)	Em (MPa)	Bending work (source: J/mm³)
40 M1	40 M1	40 M1	40 M1	40 M1
1	57,8	24,01	3582,3	29,9
2	80,5	43,74	3803,5	93,9

Test / group	fm (N/mm²)	Proportional-limit stress (MPa)	Em (MPa)	Bending work (source: J/mm³)
3	89,7	48,73	3905,3	110,7
4	125,5	48,73	5228,3	125,9
5	92,8	59,50	4189,9	20,5
6	108,6	20,95	4177,8	89
7	31,4	45,01	1861,4	69,9
Stan. deviation	29,05	12,95	937,19	36,69
Average	83,75	41,52	3821,21	77,11
40M2	40M2	40M2	40M2	40M2
1	106,8	14,12	4136,3	11,2
2	126,7	49,20	5839,0	76,9
3	97,4	45,08	5853,7	64,7
4	107,0	17,94	4755,8	15,6
5	116,4	53,00	5312,9	99,7
6	126,4	77,00	5497,3	201,3
7	87,0	37,64	4380,9	60,4
Stan. deviation	13,60	19,94	641,52	59,15
Average	109,67	42,00	5110,84	75,68
40M3	40M3	40M3	40M3	40M3
1	137,9	56,70	6160,4	99,3
2	132,4	50,80	6404,1	76,5
3	127,4	50,30	5541,2	86,8
4	104,2	44,88	5122,2	73,2
5	48,8	43,70	3847,1	99,8
6	117,8	49,31	5272,8	84,3
7	124,4	51,50	5717,2	87,9
Stan. deviation	28,18	4,02	774,76	9,44
Average	113,27	49,60	5437,86	86,83

S7. Parent-Beam Means Used for Laboratory Inference

Table S7. One analysis record per full-size parent beam for laboratory-property inference. The group label is the parent-beam preparation group; coupons were conditioned according to the laboratory protocol before testing.

Connector	Parent group	Parent ID	fm (MPa)	PL (MPa)	Em (GPa)	Work index	Density (kg/m³)
R	15	R1	91.70	37.49	10.585	14.86	445.79
R	15	R2	67.30	26.07	9.259	10.64	475.61
R	15	R3	84.77	29.04	10.491	11.00	449.88
R	20	R1	198.90	95.90	10.628	165.60	504.50
R	20	R2	71.15	33.41	3.578	60.93	363.95
R	20	R3	111.38	43.43	5.388	74.44	367.53
R	40	R1	99.09	49.04	4.265	112.20	328.64
R	40	R2	107.77	43.11	4.506	90.39	347.90
R	40	R3	107.63	44.87	4.860	77.80	322.98
M	15	M1	70.93	24.14	9.008	9.51	415.33
M	15	M2	68.83	14.73	7.928	5.70	362.64
M	15	M3	64.09	24.97	8.186	9.50	426.38
M	20	M1	136.77	54.05	6.943	90.09	410.98
M	20	M2	128.98	66.92	6.588	129.15	406.68
M	20	M3	150.56	74.27	6.782	154.54	421.20
M	40	M1	83.75	41.52	3.821	77.11	353.46
M	40	M2	109.67	42.00	5.111	75.68	359.88
M	40	M3	113.27	49.60	5.438	86.83	364.94

S8. Full-Size Analysis Dataset

Table S8. Full-size specimen-level data used for direct-group and sensitivity analyses. Density is the mapped parent-beam mean from the source physical-property tables.

Specimen	Connector	Nominal group	History	Measured MC (%)	Fmax (N)	Density (kg/m³)
1	M	40	40→20	21.9	10836	353.46
2	M	40	direct	33.6	11703	359.88
3	M	40	direct	47.3	9608	364.94
4	M	20	direct	20.9	13602	410.98

Specimen	Connector	Nominal group	History	Measured MC (%)	Fmax (N)	Density (kg/m³)
5	M	20	direct	22.8	10454	406.68
6	M	20	direct	19.3	13106	421.20
7	M	15	direct	21.8	14015	415.33
8	M	15	direct	16.2	14149	362.64
9	M	15	15→20	19.0	10320	426.38
10	R	40	40→20	22.7	9236	328.64
11	R	40	direct	23.2	10516	347.90
12	R	40	direct	29.5	9040	322.98
13	R	20	direct	18.5	10502	504.50
14	R	20	direct	19.7	9546	363.95
15	R	20	direct	20.3	11269	367.53
16	R	15	direct	15.2	14582	445.79
17	R	15	direct	15.8	13860	475.61
18	R	15	15→20	18.7	13726	449.88

S9. Statistical Diagnostics and Robustness Checks

Table S9. Principal statistical diagnostics and robustness analyses reported in the manuscript.

Analysis	Statistic	p / check	Interpretation
Full-size interaction	$F(2,8)=0.985$	$p=0.414$	Interaction not supported; additive main-effects model used.
Full-size moisture	$F(2,10)=12.289$	$p=0.0020$; permutation $p=0.0030$	partial $\eta^2=0.711$
Full-size connector	$F(1,10)=2.852$	$p=0.122$; permutation $p=0.123$	partial $\eta^2=0.222$
Full-size rank moisture	$H(2)=8.381$	$p=0.0151$	Holm MW: 15–20 $p=0.0286$; 15–40 $p=0.0571$; 20–40 $p=0.476$
Actual MC vs Fmax	Spearman $\varrho=-0.591$	$p=0.0260$	Direct specimens, n=14
Density vs Fmax	Spearman $\varrho=0.442$	$p=0.1138$	Direct specimens, n=14
Lab fm moisture	$F(2,14)=7.045$	$p=0.0076$; permutation $p=0.0044$	partial $\eta^2=0.502$; KW $p=0.0055$
Lab PL moisture	$F(2,14)=9.094$	$p=0.0029$; permutation $p=0.0012$	partial $\eta^2=0.565$; KW $p=0.0030$

Analysis	Statistic	p / check	Interpretation
Lab Em moisture	F(2,14)=12.943	p=0.00066; permutation p=0.0010	partial η^2=0.649; KW p=0.0061
Lab work moisture	F(2,14)=22.729	p=0.000040; permutation p=0.00020	partial η^2=0.765; KW p=0.0031
Lab density vs Em	Spearman ϱ=0.874	p=2.1×10⁻6	n=18 parent beams
Lab density vs fm	Spearman ϱ=0.003	p=0.990	n=18 parent beams
Residual diagnostics: fm	Shapiro p=0.0024; Levene p=0.181	–	Non-normality motivates permutation/rank robustness checks.
Residual diagnostics: PL	Shapiro p=0.0120; Levene p=0.436	–	
Residual diagnostics: Em	Shapiro p=0.0026; Levene p=0.250	–	
Residual diagnostics: work	Shapiro p=0.0319; Levene p=0.409	–	

Statistical note: Because the archived source data are small and partly unbalanced, permutation and rank-based tests were used as robustness checks. These do not create additional replication; the independent unit remains the full-size parent beam. No adjustment or imputation was applied to the four single-specimen moisture-history cases.
